# EEG-based sensory testing reveals altered nociceptive processing in elite endurance athletes

**DOI:** 10.1007/s00221-022-06522-4

**Published:** 2022-12-15

**Authors:** Malte Anders, Elias Dreismickenbecker, Johannes Fleckenstein, Carmen Walter, Elena K. Enax-Krumova, Michael J. M. Fischer, Matthias Kreuzer, Sebastian Zinn

**Affiliations:** 1 Clinical Development and Human Pain Models, Fraunhofer Institute for Translational Medicine and Pharmacology ITMP, Theodor-Stern-Kai 7, 60596 Frankfurt, Germany; 2 Center for Pediatric and Adolescent Medicine, Childhood Cancer Center, University Medical Center Mainz, 55131 Mainz, Germany; 3 Department of Sports Medicine and Exercise Physiology, Institute of Sports Sciences, Goethe University, 60596 Frankfurt, Germany; 4 Department of Neurology, BG University Hospital Bergmannsheil gGmbH Bochum, Ruhr University Bochum, 44789 Bochum, Germany; 5 Center of Physiology and Pharmacology, Medical University of Vienna, 1090 Vienna, Austria; 6 Department of Anesthesiology and Intensive Care, School of Medicine, Technical University of Munich, 81675 Munich, Germany; 7 Department of Anesthesiology, Intensive Care Medicine and Pain Therapy, Goethe University, University Hospital Frankfurt, 60590 Frankfurt, Germany

**Keywords:** EEG, Pain, Nociception, Elite endurance athletes, Conditioned pain modulation, Event-related spectral perturbation

## Abstract

Increased exercise loads, as observed in elite athletes, seem to modulate the subjective pain perception in healthy subjects. The combination of electroencephalography (EEG) and standardized noxious stimulation can contribute to an objective assessment of the somatosensory stimulus processing. We assessed the subjective pain ratings and the electroencephalogram (EEG)-based response after standardized noxious mechanical and thermal stimuli as well as during conditioned pain modulation (CPM) in 26 elite endurance athletes and compared them to 26 recreationally active controls. Elite endurance athletes had consistently stronger somatosensory responses in the EEG to both mechanical and thermal noxious stimuli than the control group. We observed no significant group differences in the subjective pain ratings, which may have been influenced by our statistics and choice of stimuli. The CPM testing revealed that our conditioning stimulus modulated the subjective pain perception only in the control group, whereas the EEG indicated a modulatory effect of the conditioning stimulus on the spectral response only in the athletes group. We conclude that a higher activation in the cortical regions that process nociceptive information may either be an indicator for central sensitization or an altered stimulus salience in the elite endurance athletes’ group. Our findings from our CPM testing were limited by our methodology. Further longitudinal studies are needed to examine if exercise-induced changes in the somatosensory system might have a critical impact on the long-term health of athletes.

## Introduction

Elite athletes experience pain with some regularity. They have a very high lifetime prevalence of up to 84% for chronic pain syndromes including lower back pain (Fett et al. 2017; Farahbakhsh et al. 2018), with a broad variety of biopsychosocial factors playing a role even at early stages in their careers (Bumann et al. 2020). One of the possible risk factors for the chronification of pain may be an altered nociceptive processing (Roussel et al. 2013). To evaluate if endurance exercise influences the nociceptive processing and pain perception of elite athletes, it is important to understand how pain is defined, assessed, and quantified.

Pain by its definition is a personal experience depending on biological, psychological, and social factors and, thus, is influenced by subjective factors (Raja et al. 2020). The assessment of nociceptive processing is tricky; it aims to objectively quantify pathophysiological changes besides assessing psychosocial variates (Sommer 2016; Treede et al. 2019). Subjective pain testing is the gold standard in research, e.g., via questionnaires such as the McGill pain questionnaire (Main 2016), via quantitative sensory testing (QST) (Rolke et al. 2006), or with paradigms testing the conditioned pain modulation (CPM) (Nir and Yarnitsky 2015). In addition, electroencephalography (EEG)-based cortical-evoked potentials in response to noxious stimuli have been introduced as promising tools (van den Broeke et al. 2015; Özgül et al. 2017; Hüllemann et al. 2019; Fabig et al. 2021). Nociceptive testing using EEG can be carried out in a non-verbal population such as newborn infants (Hartley et al. 2017) or in animals (Murrell and Johnson 2006), and advances in computerized analytics of the EEG, like the analysis of the event-related spectral perturbation (ERSP) and the inter-trial coherence (ITC), allow for an in-depth analysis of event-related EEG data. While subjective pain ratings give an insight into the subjective sensory response of the body to nociception (“pain perception”), certain methods of neuroimaging e.g., high-density multi-channel EEG recordings combined with standardized noxious stimulation, enable a different and not invariably correlated analysis: the activation of the cortical regions in the brain, which are called the “pain matrix”, to noxious stimuli. The activation of these cortical structures as measured by the EEG is not only dependent on the perceived painfulness of the stimulus, but also on the stimulus salience of an individual, i.e., the significance the participant is directing towards the stimulus (Iannetti et al. 2008; Legrain et al. 2011). It is thus usefully extending the conventional approach of only testing the subjective pain ratings.

The differences in pain perception between elite athletes and a normally active population have been studied extensively, although no studies have integrated the EEG into their testing paradigm. In the literature, a higher pain tolerance of elite athletes is concluded and higher pain thresholds are suggested (Tesarz et al. 2012). Recent research seems to confirm these findings (Geva and Defrin 2013; Tesarz et al. 2013; Pettersen et al. 2020). Furthermore, the type of sports, e.g., strength versus endurance, does seem to play a major role in the exact changes in pain perception (Assa et al. 2019). Two studies evaluated the acute effects of exercise on nociceptive processing in trained athletes using functional magnetic resonance imaging (fMRI) (Scheef et al. 2012; Geisler et al. 2021), with different aims: the first study researched the acute short-term effect of endurance exercise on the pain response as measured in the fMRI (Scheef et al. 2012), while the other researched the long-term neuronal alterations as a result of heavy endurance exercise (Geisler et al. 2021). Scheef et al. concluded that acute endurance exercise in elite athletes reproducibly suppressed the activation of pain-induced processes in different cortical brain regions that are responsible for nociceptive processing, alongside with elevated levels of antinociceptive endogenous opioid neuropeptides. Geisler et al. concluded that in the long term, high training levels of endurance sports also seem to suppress the activation of these cortical structures, compared to a sedentary control group. Although the fMRI excels in providing such detailed spatial information, its temporal resolution of these processes is significantly inferior to the EEG (Cohen 2011).

As the EEG provides information with a high-temporal-resolution about the neural processing of nociception, we analyzed fast-acting time-locked nociceptive-related processes after standardized noxious stimulation of trained endurance athletes, as compared to non-elite, recreationally active controls. We also examine if there are differences in the endogenous pain modulation capacities as assessed by CPM, both via the EEG and subjective pain scores. We aimed at analyzing if our groups differed in their conventional subjective pain ratings to our standardized nociceptive stimuli and if those differences were represented in a similar fashion in their activation of the pain matrix as expressed by our EEG data. In contrast to the existing neuroimaging studies of elite endurance athletes, we aimed at capturing short-term processes after brief noxious stimulation in the range of milliseconds, which cannot be reliably captured by the fMRI. We hypothesize that there are long-term modulatory effects of elite endurance sports on the cortical regions that process nociception, which can be uncovered using neuroimaging tools but not by subjective pain testing alone.

## Results

### Participants: total numbers and anthropometric data

We recorded and analyzed data from 26 elite endurance athletes and compared them to 26 normally active controls. Their anthropometric and sport-specific data as well as data regarding the pain history of the groups are outlined in Table 1. The athletes engaged in rowing (12 participants), triathlon (9 participants), speed skating (3 participants) and running (2 participants). The hourly weekly training load included endurance, weight training and circuit training. Our athletes had a significantly higher training load, a significantly lower resting heart rate and reported more frequent suffering from pain levels in the past 3 months. Both study groups did not differ in their quality of life as assessed by the Veterans RAND 12 Item Health Survey (VR-12) global health questionnaire, a questionnaire for the self-evaluation of one’s health-related quality of life.

**Table 1:** Anthropometric data of the participants included in our study, VAS scores for the standardized noxious mechanical and heat stimulation, and questionnaire responses

	Active controls	Elite endurance athletes	Statistics
Number and sex of participants	26 (15 male, 11 female)	26 (15 male, 11 female)	*n.a.*
Age	25.5 [24.6; 27.1] years	26.1 [23.5; 29.7] years	*n.s.* *p* = 0.71
Weekly training load	4 [2; 6] hours	20 [18; 24] hours	*p < 0.001
Question: for how many years have you been training for more than 15 h/week?	0 [0; 0]	8 [5; 10]	*p < 0.001
Heart rate after the submaximal endurance test	136 [120; 148]	90 [80; 108]	*p < 0.001
Resting heart rate	64 [60; 72]	48 [44; 56]	*p < 0.001
Pcs-12 scores	61.8 [59.0; 62.2]	61.7 [57.0; 63.2]	*n.s.* *p* = 0.93
Mcs-12 scores	39.8 [36.0; 41.6]	41.0 [38.7; 43.5]	*n.s.* *p* = 0.17
Mechanical pain during PEP	12 [5; 20]	11 [7; 18]	*n.s.* *p* = 0.95
Heat pain during CHEPS	21 [13; 34]	17 [9; 33]	*n.s.* *p* = 0.30
CPM + pinprick: before water bath	19 [11; 27]	16 [6; 22]	*n.s.*
CPM + pinprick: during water bath	13 [9; 18]	14 [5; 24]	*n.s.*
CPM + pinprick: after water bath	20 [8; 31]	16 [4; 23]	*n.s.*
Conditioning stimulus (8 °C water bath): initial pain intensity after inserting the foot	28 [15; 40]	15.5 [6; 29]	*p = 0.026
Vas: average pain intensity in the past 3 months (0–100)	2 [0; 5]	14 [3; 34]	*p = 0.007
Vas: current pain level at rest	0 [0; 0]	1 [0; 3]	*p = 0.007
Question: have you experienced pain that persisted/recurred for more than 3 months (ICD-11 definition of chronic pain)?	Yes: *n* = 2 No: *n* = 24	Yes: *n* = 5 No: *n* = 21	*n.s.* *p* = 0.22
Question: have you experienced a sports-related injury in the past year, and can you specify the body region?	Yes: *n* = 4 No: *n* = 22	Yes: *n* = 13 No: *n* = 13	*p = 0.007
Question: is your current pain sports-associated?	Yes: *n* = 4 No: *n* = 22	Yes: *n* = 19 No: *n* = 7	*p < 0.001
Question: are you doing sports while suffering from pain?	Yes: *n* = 6 No: *n* = 20	Yes: *n* = 19 No: *n* = 7	*p < 0.001

The data are presented as median values with the 25% and 75% percentiles stated in square brackets. An asterisk in the statistics column depicts a *p* value smaller than 0.05, “*n.s.*” depicts a *p* value greater than 0.05 and “*n.a.*” depicts that we did not calculate statistics for the comparison

*PCS* physical component score, *MCS* mental component score, *VAS* visual analog scale, *n.s.* not significant, *n.a.* not applicable, *CHEPS* contact heat-evoked potentials

### Visual analogue scale (VAS): subjective pain ratings to standardized noxious stimuli

Both groups did not differ in their subjectively perceived pain intensity following mechanical or heat noxious stimuli (see Table 1).

The subjective pain ratings of the test stimulus were affected differently by the conditioning stimulus in both groups (see Table 1). In the controls’ group, the conditioning stimulus significantly lowered the subjective pain ratings to the test stimulus (see Table 2). After the conditioning stimulus was removed, the pain ratings significantly increased back to baseline levels. We did not observe a significant decrease or increase of the subjective pain ratings to the test stimulus because of the conditioning stimulus in the elite endurance athletes’ group between any of the conditions.

**Table 2:** Intra-group statistical testing of the relative change in subjective pain ratings of the test stimulus difference between the CPM conditions before vs. during, during vs. after, and before vs. after the administration of the conditioning stimulus

	Recreationally active controls	Elite endurance athletes
Intra-group testing: Friedman’s test: CPM + pinprick before vs. during vs. after	*p = 0.001	*n.s.* *p* = 0.13
Intra-group post hoc testing: before vs. during	*p = 0.007	Not tested
Intra-group post hoc testing: during vs. after	*p = 0.003	Not tested
Intra-group post hoc testing: before vs. after	*n.s.* *p* = 0.96	Not tested

For the intra-group testing, we show the Friedman’s/multcompare statistics and the *p* value. An asterisk depicts statistical significance

*CPM* conditioned pain modulation, *n.s.* not significant, *n.a.* not applicable

### Mechanical pain: pinprick-evoked potentials (PEP) as the spectral perturbation

**Fig. 1﻿:**
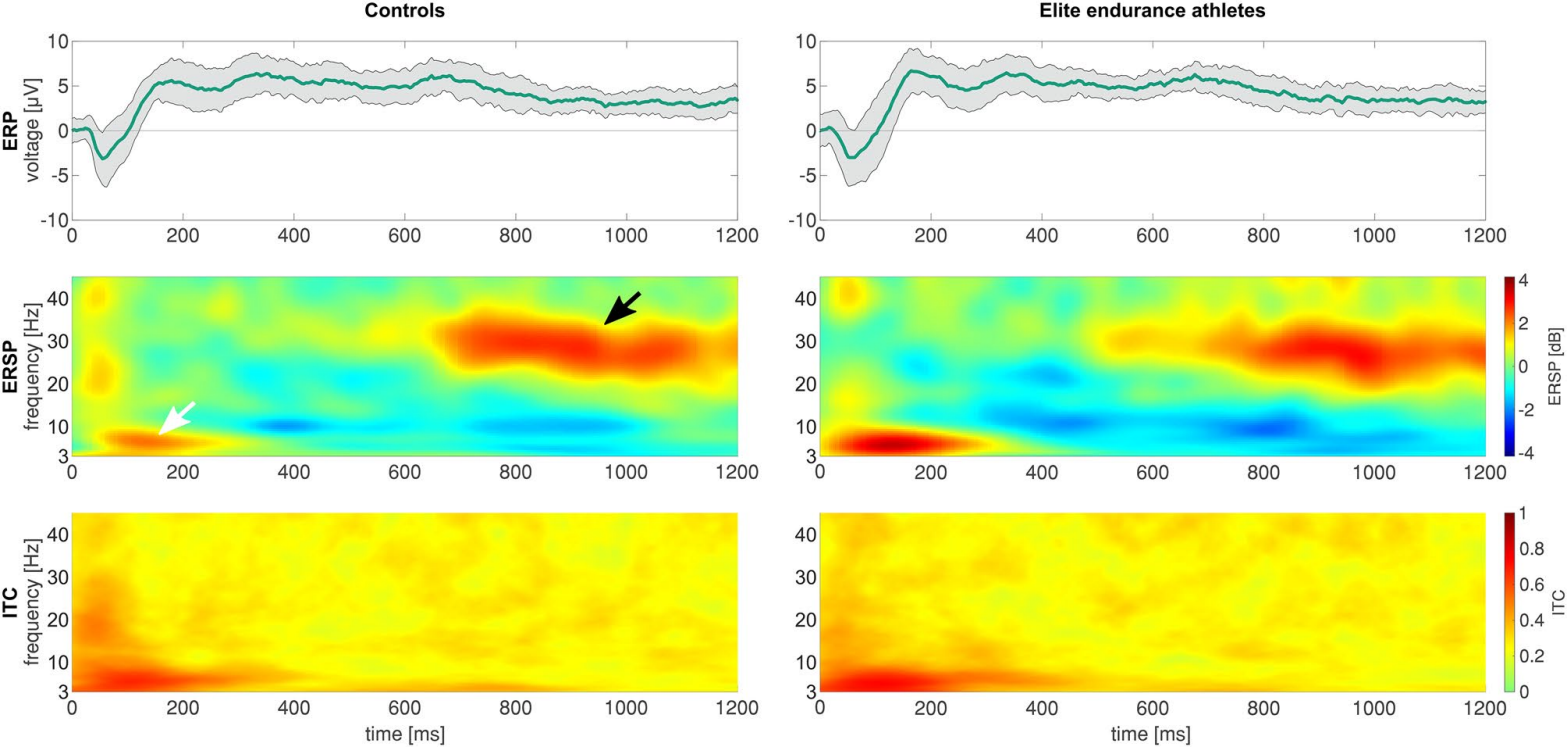
Raw EEG data and spectral changes of the pinprick-evoked potentials in the controls and elite endurance athletes at electrode Cz. Upper row: event-related potential (ERP). Original EEG data in the amplitude-time spectrum after pinprick stimulation for both the controls and the elite endurance athletes. Traces are the means across all 12 trials per participant and all 26 participants. The shaded area indicates the standard deviations of the 26 participants. Middle row: Average event-related spectral perturbation (ERSP), i.e., the spectral changes over time. The white arrow indicates the N2P2 response, while the black arrow indicates a response not visible in the ERP analysis. Lower row: inter-trial coherence (ITC) or phase locking factor (PLF), i.e., the spectral synchronization among the trials

**Fig. 2﻿:**
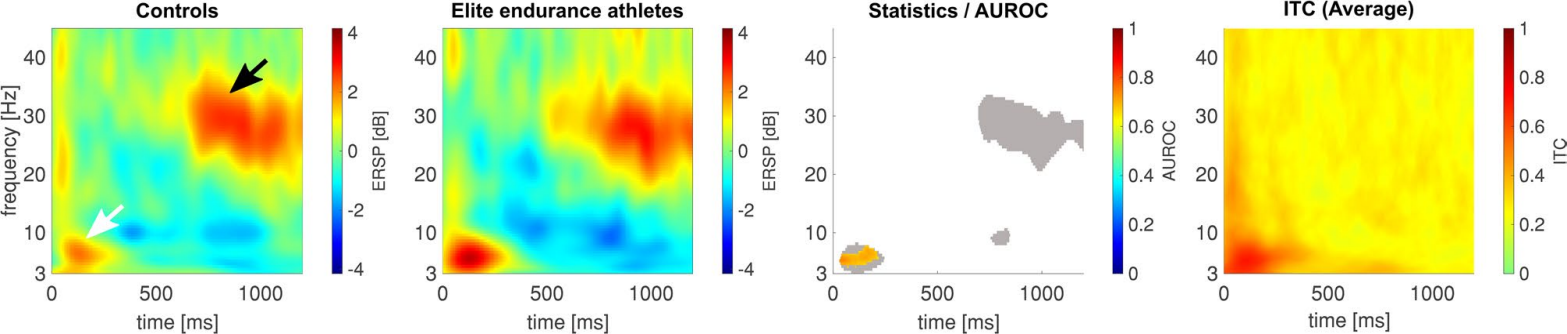
Elite endurance athletes show stronger pinprick-evoked potentials at electrode Cz, compared to controls. Panel 1 shows the event-related spectral perturbation of the controls, likewise, panel 2 shows that of the elite endurance athletes. Third panel: The statistical comparison includes both a common non-parametric statistical testing with a cluster-based correction for multiple comparisons, and the AUROC effect size. Pixels that are significantly different are colored red/orange or blue, according to the c-axis next to the image, which indicates the AUROC effect size of the comparison. The gray-shaded area in the statistics image indicates that the accompanying pixel in the ERSP in either one of the groups exceeds a [2 dB; − 2 dB] range; areas of interest for further analysis are highlighted in panel 1 with a white (early low-frequency response) and black arrow (late high-frequency response). The fourth panel shows the average ITC calculated for all 52 participants to help the reader to identify the area where the N2P2 component is commonly found

We show the raw event-related EEG in the amplitude-time spectrum and the event-related spectral perturbation (ERSP) in Fig. 1 and our ERSP-based group comparison of the pinprick stimuli in Fig. 2. On average, athletes had a significantly higher response to mechanical stimulation in the area with the highest degree of phase locking (white arrow in Fig. 2) than the control group, indicating that they elicited a higher EEG-based response to noxious mechanical stimuli. The maximum average ITC value of 0.71 was found in that area. The maximum ERSP values were 3.60 dB (athletes at 5.44 Hz and 129 ms) and 2.18 dB (controls at 5.92 Hz and 129 ms). The ERSP values between both groups were significantly different. The maximum AUROC value was 0.74 [0.61; 0.87] at 27 ms and 5.44 Hz. We classified our AUROC values according to a traditional point system with a value of 0.74 indicating a fair effect.

There was no significant difference in the later high-frequency response with a low degree of phase locking between the two groups (black arrow in Fig. 2). The maximum ERSP value was 3.06 dB (at 26.40 Hz and 969 ms) in the athletes’ group vs. 2.81 dB (at 28.35 Hz and 910 ms) in the controls’ group.

### Heat pain: contact heat-evoked potentials (CHEPS) as the spectral perturbation

**Fig. 3:**
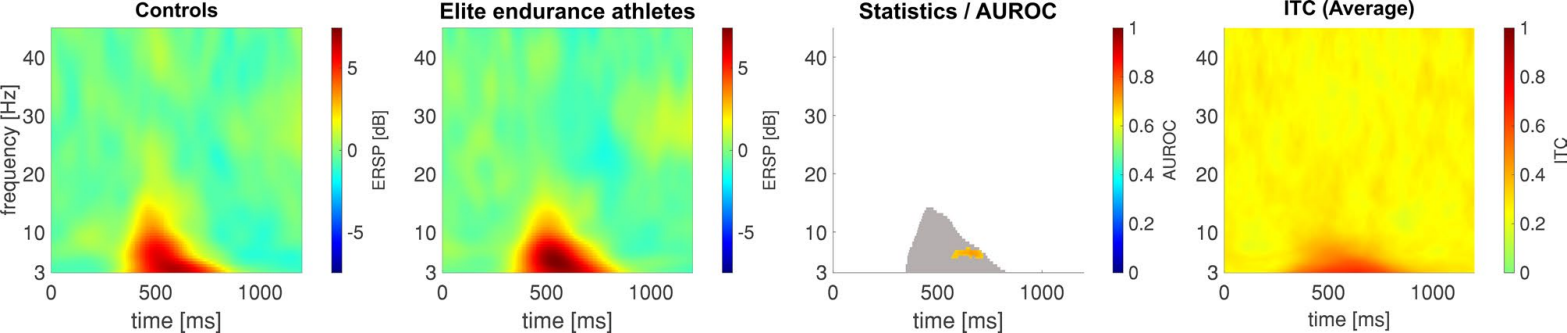
Elite endurance athletes show stronger contact-heat-evoked potentials at electrode location Cz, compared to controls. Panel 1 shows the event-related spectral perturbation of the controls, likewise, panel 2 shows that of the elite endurance athletes. Third panel: The statistical comparison includes both a common non-parametric statistical testing with a cluster-based correction for multiple comparisons, and the AUROC effect size. Pixels that are significantly different are colored red/orange or blue, according to the c-axis next to the image, which indicates the AUROC effect size of the comparison. The gray-shaded area in the statistics image indicates that the accompanying pixel in the ERSP in either one of the groups exceeds a [2 dB; − 2 dB] range. The fourth panel shows the average ITC calculated for all 52 participants to help the reader to identify the area where the N2P2 component is commonly found

In Fig. 3, elite endurance athletes elicited a higher EEG-based response to noxious contact heat stimuli in the EEG. The maximum ERSP value for this response was 6.71 dB at 3.49 Hz and 582 ms in the control group and 7.45 dB at 4.46 Hz and 547 ms in the elite endurance athletes’ group. This response had the highest average ITC, with a maximum value of 0.67. There was a significant difference with a maximum AUROC value of 0.73 [0.57; 0.87] at 6.41 Hz and 656 ms, indicating a fair effect between both groups.

### Conditioned pain modulation (CPM): assessing the endogenous pain inhibition mechanisms

**Fig. 4:**
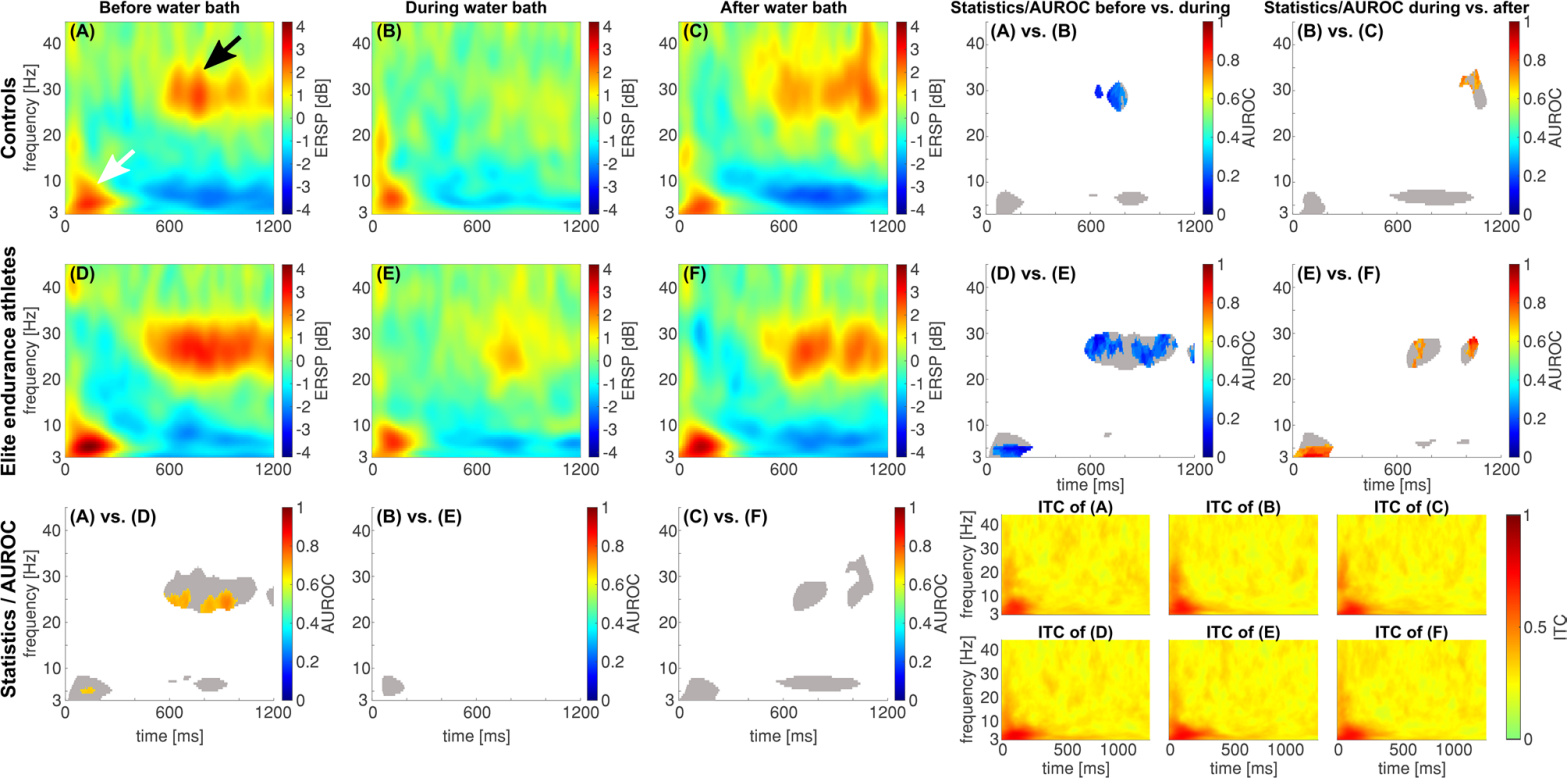
The conditioning stimulus during conditioned pain modulation testing affects the N2P2 component only in the elite endurance athletes, but not in the controls. A noxious cold water bath at 8 °C was used as the conditioning stimulus (CS). The event-related spectral perturbation (ERSP) of pinprick-evoked Potentials (PEP as the test stimulus) plotted before, during and after the conditioning stimulus for the controls (**A**, **B** and **C**) as well as for the elite endurance athletes (**D**, **E** and **F**). The right panels show a statistical comparison between the conditions before vs. during and during vs. after for both groups; the bottom panel shows a group comparison between the groups for each condition. The gray-shaded area in the statistics image indicates that the accompanying pixel in the ERSP in either one of the groups, or conditions, exceeds a [2 dB; − 2 dB] range, and is considered an EEG response to the stimulus. Furthermore, our areas of interest are highlighted in panel 1 with a white and black arrow (white arrow for the early low-frequency response, black arrow for the late high-frequency response). The statistical comparison includes both a common non-parametric statistical testing (paired for intra-group testing and unpaired for inter-group testing) with a cluster-based correction for multiple comparisons and the AUROC effect size. Pixels that are significantly different are colored red/orange or blue, according to the c-axis next to the image, which indicates the AUROC effect size. The lower right panels show the respective inter-trial coherence (ITC) for each condition and group

The low-frequency, highly phase-locked response to the test stimulus was only significantly affected by the conditioning stimulus in the elite endurance athletes’ group, but not in the control group, as shown in Fig. 4. During the conditioning stimulus in the elite endurance athletes’ group, the maximum value of this highly phase-locked response was significantly reduced from 4.19 dB in (D) to 2.81 dB in (E) and increased to 3.76 dB thereafter (F). The minimum value of the AUROC was 0.08 [0; 0.19] at 4.95 Hz and 187 ms (D vs. E), while the maximum AUROC value was 0.92 [0.81; 1] at 3.0 Hz and 74 ms (E vs. F), both indicating an excellent effect between the two conditions.

In the control group, the changes in the ERSP of the low-frequency, highly phase-locked response were not statistically significant during our CPM testing. The maximum ERSP value decreased from 2.84 dB in (A) before the application of the cold pressor task to 2.55 dB in (B) during the cold pressor task and increased to 2.58 dB in (C) thereafter.

The maximum ERSP values of the low-frequency, highly phase-locked response during the before-condition were 2.84 dB at 4.95 Hz at 129 ms in the control group, and 4.19 dB at 4.95 Hz and 133 ms in the elite endurance athletes’ group. The difference between these was significant; the maximum AUROC value was 0.68 [0.53; 0.82] at 4.95 Hz and 133 ms between the groups (A vs. D), indicating a poor effect.

Our conditioning stimulus affected the response in the higher frequency regions in both groups (black arrow). The maximum ERSP values before the water bath were 2.62 dB for the controls and 2.99 dB for the elite endurance athletes. The difference between these was significant; the maximum value of the AUROC was 0.75 [0.61; 0.89] at 23.96 Hz and 930 ms between the groups before the cold pressor task (A vs. D), indicating a fair effect.

The maximum ERSP values of the high-frequency response during the ongoing cold pressor task decreased to 0.75 dB in the controls and 1.73 dB in the elite endurance athletes. The decrease of both responses was significant in both groups, with a minimum AUROC effect size of 0.12 [0; 0.23] at 29.80 Hz and 672 ms in the control group (A vs. B, good effect) and a minimum AUROC effect size of 0.08 [0; 0.19] at 28.83 Hz and 625 ms in the elite endurance athletes (D vs. E, excellent effect).

One minute after the cold pressor task, the ERSP of the high-frequency response increased to a maximum value of 2.36 dB in the controls and 2.62 dB in the elite endurance athletes. This increase is significant in both groups, with a maximum AUROC effect size of 0.77 [0.58; 0.92] at 31.76 Hz and 973 ms in the controls (B vs. C, fair effect) and a maximum AUROC effect size of 0.88 [0.77; 1] at 27.86 Hz and 1020 ms in the elite endurance athletes (E vs. F, good effect).

## Discussion

### Subjective pain perception

The consensus in the literature suggests that with growing age, the pain thresholds of elite athletes increases and pain is increasingly tolerated, i.e., the subjective response to a noxious stimulus is silenced (Pettersen et al. 2020). From our data and for our noxious stimulation methods, we could not conclude this in our cohort of elite athletes with a median age in their mid-twenties. The subjective pain perception of noxious events in our cohort of 18–35-year-old participants did not differ between participants with a recreational training level and the elite level when using brief, noxious, tonic stimulation. As this contradicts the findings of most of the available literature, other factors may have played a role in our study: our small sample size per group, a high interindividual variability, and our choice of stimuli may have prevented us from unmasking differences in the subjective pain perception. After all, a significant difference in the initial pain rating of the conditioning stimulus indicated that our elite athletes are, at least in the case of a noxious cold water bath, more pain-resilient than the controls. Hence, our brief, tonic, noxious stimulation may be a good way to research the nociceptive processing in the EEG, but not a suitable way to test for differences in the subjective pain ratings, for which a full somatosensory testing panel such as quantitative sensory testing (QST) would be the more appropriate research method.

### Differences in EEG-based processing of standardized noxious stimuli during the resting state

A higher activation in an area with a high degree of phase locking in the elite endurance athletes’ group may be interpreted as a sign of a central sensitization to noxious stimuli, i.e., the stimulus activates the central processing units in the S2 region of the brain in a stronger fashion in the elite endurance athletes’ group than in the control group. Pinprick-evoked potentials (PEPs) have been demonstrated to be an objective tool to quantify the effect of an experimentally induced secondary mechanical hyperalgesia and have been suggested to be a viable diagnostic tool for mechanical hyperalgesia for patients with a presumed central sensitization (Iannetti et al. 2013; van den Broeke et al. 2015; van den Broeke et al. 2017). From our data, a central sensitization or a hyperalgesia could not be concluded from the subjective pain ratings. Thus, although the theory of a central sensitization cannot be confirmed by the subjective pain ratings, there are some methodological aspects regarding the type of stimuli, the sample size, and the interindividual variability that need to be accounted for as discussed in the previous paragraph. Hence, we still propose a central sensitization of our elite endurance athletes as a possible reason for the significant increase in the EEG response, which may not be unmasked by our subjective pain testing due to methodological limitations. This theory is also backed up by the pain history in Table 1. Although there was no difference in the recurrence of chronic pain in both groups, our athletes still suffered from sport-associated pain states more frequently. Recurring pain has been shown to induce neuroplastic changes in both the brain and the spinal cord, and the literature clearly proves that it leads to central sensitization (Latremoliere and Woolf 2009; Nijs et al. 2021).

Another possible explanation for the higher EEG-based activation of the athletes is that ERPs or the corresponding N2P2-related component in the ERSP, as elicited by transient nociceptive stimuli, are mostly determined by two factors: the painfulness of the stimulus, and by the stimulus salience (Iannetti et al. 2008; Ronga et al. 2013). The salience is the property of a stimulus of how much it can capture attention, i.e., how much focus the participant will pay to the stimulus. If we account for the fact that elite endurance athletes perceived the stimulus as equally as painful as the control group, another likely explanation would be an increased salience to noxious mechanical and heat stimulation, as represented by the increased activation of the N2P2-representing component in the ERSP of the EEG. This may indicate as some sort of “priming” of the athletes to noxious events and subsequently, pain, to which they are somewhat used to due to their sports career (Fett et al. 2017; Farahbakhsh et al. 2018; Bumann et al. 2020). Eventually, this process may lead to coping strategies such as suppressing the subjective pain response, as shown by other studies (Pettersen et al. 2020).

Interestingly, a recent study that researched the pain perception of elite endurance athletes using the fMRI seems contradicting to our data (Geisler et al. 2021), as for elite endurance athletes compared to a sedentary control group, their data revealed a significantly reduced response to a noxious heat stimulus in cortical regions such as the insula and the anterior cingulate cortex. These regions are usually also captured by our EEG methodology. However, not only is there a difference in taking either a sedentary versus a normally active control group and some individual researchers even recommend using only physically/normally active control groups (Booth and Lees 2006; Buford and Manini 2010). A sedentary lifestyle has been shown to be a factor in the development of chronic pain (Senba and Kami 2017), which would further add confounding factors to the interpretation of our data. As stated in the introduction, the fMRI and the EEG also excel in different areas regarding spatial and temporal resolution. Using the fMRI, a width of the BOLD response of ~ 3 s and a peak that occurs ~ 5–6 s after the onset of a brief stimulus are common (Kim et al. 1997; Glover 2011), so that the very early processes in the range of milliseconds after a brief noxious stimulus that we captured in this manuscript cannot be analyzed. Unsurprisingly, the researchers thus also relied on long-acting noxious thermal stimuli (20 s), which are vastly different from the brief stimuli applied in this manuscript. A combined fMRI/EEG approach is an interesting approach for future research about the differences in processing of short- and long-acting noxious stimuli in endurance athletes.

For the brief noxious stimuli in our data, both a presumed central sensitization and/or an altered stimulus salience were a likely explanation for the observed statistical differences. Our conclusion is limited by the methodological choices, as the exact reason for the altered response cannot be answered by only using EEG data. Hence, future studies may consider our postulated explanations in their research, e.g., by incorporating the combination of the EEG and fMRI into the study design.

### Conditioned pain modulation

A recent meta-analysis about conditioned pain modulation in elite endurance athletes (McDougall et al. 2020) concluded that aggregated results, despite a higher nominal number of studies reporting higher CPM capacities in athletes, do not favor a significant difference. While a possible correlation between training hours and CPM capacities is suggested, the meta-analysis also points out the methodologically low quality of several studies. The conclusion is, however, further supported by preclinical studies (Sluka et al. 2018), but it is not in-line with our findings. We will now discuss that this is probably also due to methodological issues but also offer a hypothetical explanation for our data. Overall, as stated in the meta-analysis, higher quality CPM data will be needed to analyze the full extent of the modulatory effects of endurance sports on endogenous pain inhibition. Our following discussion about our own methodological issues may be considered by future studies to achieve the necessary higher quality in CPM testing of elite endurance athletes.

In our data, the conditioning stimulus significantly reduced the subjective pain ratings of the test stimulus only in the controls’ group. By design of the CPM paradigm, this is the expected effect: the pain rating of a test stimulus is significantly reduced due to the activation of the endogenous pain inhibitory system by a conditioning stimulus (Nir and Yarnitsky 2015). We observed no such significant effect in the elite endurance athletes’ group. This may at least partially be explained by the perceived painfulness of the conditioning stimulus: our data in Table 1 shows that, although perceived as painful by both groups, the stimulus is perceived as significantly less painful in the elite endurance athletes’ group. This implies that their endogenous pain inhibitory system is less activated. Our analysis may thus be methodologically constrained by this factor, as we did not equally activate the endogenous pain inhibitory system. Using a constant stimulus energy, i.e., the same water bath temperature for both groups, this result may be expectable in elite endurance athletes: the available literature about pain thresholds in elite endurance athletes reports higher pain thresholds than in normally active controls (Pettersen et al. 2020). To achieve a comparable level of conditioning pain levels, an adaption of the temperature of the water bath to a target pain score may have been necessary. Another possible methodological limitation could also be the use of a 512 mN pinprick as a test stimulus, as this has not been reliably tested in the literature.

A different possible explanation is the pain history of our athletes’ group: our data in Table 1 indicated that our athletes, as compared to our controls, are significantly more affected by sports-related injuries and pain and show a significantly higher current pain intensity as well as a significantly higher average pain intensity in the past 3 months. In addition, as discussed above, a central sensitization may be one possible explanation for our elevated EEG response to noxious stimulation in the athletes’ group. Although the recurrence of chronic pain is not significantly higher in the athletes’ group, it has been shown that ongoing pain impairs the response to a conditioning stimulus during CPM testing (Lewis et al. 2012). This increased recurrence of pain may thus be a facilitating factor that leads to a loss of descending pain inhibitory function or an increase in descending facilitation of spinal nociceptive pathways (Bannister and Dickenson 2017), which would explain the absence of a CPM effect in our athletes group.

### Limitations

Due to our conservative statistical approach, we may have only captured strong effects between the groups and more subtle differences may need to be further investigated. As this was an explorative translational study, we did not perform a conventional a-priori sample size calculation (Bacchetti et al. 2011), as no appropriate preliminary data has been published yet. In combination with our small sample size, this may have limited us to capture statistical differences between the groups especially regarding the subjective pain ratings. Furthermore, we only analyzed the pain perception using a standardized VAS to two different stimuli and did not assess a complete somatosensory profile. For our analysis, we separated our groups into elite endurance athletes and non-athletes in a binary fashion, without taking the exact individual performance level into account. Studies that aim at determining the exact nature of this “dose–response” relationship in the future should rely on cardiopulmonary exercise testing. Furthermore, we only assessed the activity level of our control group by means of a subjective self-report in predefined categories (e.g., strength, endurance), which did not reveal the exact type and intensity of activity. Our results from the conditioned pain modulation testing are limited by our methodology.

## Methods

### Participants

The local ethics committee approved the study procedures in a written statement on 14/07/2020 (Ethics Committee of the Faculty of Psychology and Sports Sciences at Goethe University Frankfurt, reference number 2020-40). The participants received a compensation of 30€ for their successful participation. Furthermore, we conformed to the standards set by the Declaration of Helsinki and prospectively registered the study on 24/07/2020 with the WHO-approved German Clinical Trials Register (DRKS) (DRKS-ID: DRKS00022349). The study was carried out in an institution certified for QST assessment.

**Fig. 5:**
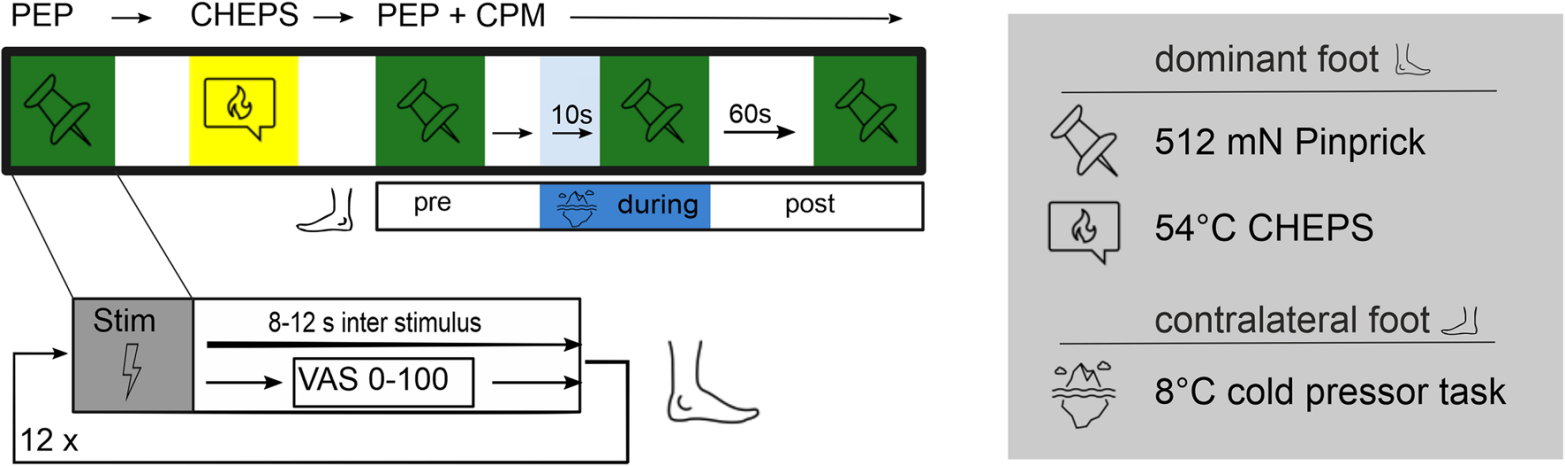
Study flow for each participant

Two groups of competitive elite endurance athletes and regularly active, non-elite controls were included in this study. To define the characteristics of an elite athlete, we followed the criteria published by Swann (Swann et al. 2015). Each participant underwent the same study flow as outlined in Fig. 5. The main inclusion criterion for the elite endurance athletes was a regular training for national or international competitions in their respective type of endurance sport with a training load of at least 15 h per week for the past 2 years. This was based on the average total training time of a German elite athlete (Breuer and Wicker 2010). In contrast, participants in our control group did not engage competitively in elite endurance sports on a national or international level. We required them to have never partaken competitively in any type of sports at elite level with a training load greater than 15 h per week. Participants in both groups reported their weekly hours of training load in terms of the American College of Sports Medicines’ definition of exercise (Pescatello and Wilkins 2014).

General inclusion criteria were a minimum age of 18 years and a maximum age of 35 years, as we relied on the QST reference values for that age group (Magerl et al. 2010). We further required no regular intake of pain medication, the absence of sensory disorders (peripheral neuropathies or neuropathic pain), the absence of depression and no intake of antidepressants, no current intake of antipsychotics and no known autoimmune diseases or pregnancy. We recruited an equal number of males and females in each group and matched those participants by age. In addition, we asked all the participants to refrain from excessive physical activity (e.g., taking part in competitions) and the intake of pain medication 24 h prior to the study. Written informed consent was sought from all the participants prior to enrollment.

### Visual analog scale (VAS)

For subjective pain ratings, we used a tablet (Apple iPad mini) with a visual analog scale app (Apple App Store: “VAS—Visual Analog Scale” by Herve Kasparian D.O. and Ghislaine Signoret D.O., Cabinet d’ostéopathie Kasparian-Signoret, France). The app consisted of a slider with a red triangle underneath and a scale ranging from no pain (left, “0”) to worst pain (right, “100”). Visual ratings corresponded to ratings from 0 (no pain) to 100 (worst pain) with graduations of 1. We presented the tablet to the participants with the slider in the left position. The numerical expression recorded could only be viewed by the examiner. According to the QST protocol (Rolke et al. 2006), the participants were instructed to move the slider to a position greater than “0” if a sensation was experienced as being painful.

### Standardized noxious stimulation

For standardized noxious stimulation, we used mechanical and thermal stimuli derived from the QST protocol (Rolke et al. 2006). We decided to use a fixed stimulus intensity on each participant by applying a fixed mechanical force or stimulating with a constant peak contact heat temperature rather than determining each participants’ individual threshold. This allowed for a robust inter-group comparison as we kept the stimulus energy constant and eliminated the influence of fluctuations in stimulus energy on our EEG response. In addition, the recent studies that published normative data for CHEPS also administered a fixed peak stimulation temperature (Granovsky et al. 2016; Jutzeler et al. 2016; Rosner et al. 2018). In order to ensure that our stimuli were perceived as painful by our healthy participants, we set the fixed stimulus intensity according to the QST reference values as described in the following paragraphs (Magerl et al. 2010). In order to avoid peripheral sensitization or stimulus wind up due to repetitive stimulation, both mechanical and contact heat stimulation were applied 12 times with a randomized or pseudo-randomized inter-stimulus interval. The number of trials in the literature ranges from 7 trials (Granovsky et al. 2016; Anders et al. 2022) up to 20 trials (Rosner et al. 2018). We stimulated an area of 9 cm × 8 cm on the dorsal area of the dominant foot and applied the stimuli in a randomized pattern across the whole stimulation area. We asked the participants to rate each stimulus approximately 2 s after onset on the VAS. We verbally announced every single stimulation with the pinprick to the participant with a trigger word, approximately 1 s prior to the stimulus. We asked the participants to keep their eyes open during the test and to avoid blinking for 2 s directly after the trigger word and to be alert on the upcoming noxious stimulus.

### Pinprick-evoked potentials (PEP)

We used a pinprick (MRC Systems, Heidelberg, Germany) with a force of 512 mN to selectively activate both A- and C-fiber mechanosensitive nociceptors (Ziegler et al. 1999; Magerl et al. 2001; van den Broeke et al. 2015). The force of 512 mN was chosen as it is above the 95% confidence interval of the mechanical pain threshold (MPT) in the QST reference data for feet stimulation in the age range of 15–35 years and should thus be perceived as painful by a healthy participant (Magerl et al. 2010). The mean values were 73.02 mN (10.97 mN; 486.09 mN) for males and 69.39 mN (9.92 mN; 485.16 mN) for females, with the 95% confidence interval in brackets (Magerl et al. 2010).

We modified the pinprick to generate a 5 V TTL trigger pulse for our EEG recordings by drilling two opposite holes into the stationary holding tube right above the moving weight, as it is described in the literature (Iannetti et al. 2013). We equipped the holes with a photodiode and a phototransistor in a way that the photoactive parts were facing each other. When we applied the pinprick onto the skin, the weight was moved upwards and disrupted the visual connection between the sensor/emitter pair. The 5 V TTL trigger pulse was then generated via an LM393 (Texas Instruments, Dallas, United States of America) and an ATmega32U4 (Microchip, Chandler, United States of America). We programmed the Integrated Circuit (IC) using the Arduino IDE (Arduino, Somerville, United States of America). We randomized the inter-stimulus interval between 8 and 12 s.

### Contact heat-evoked potentials (CHEPS)

We again stimulated the dorsal area of the dominant foot and applied thermal stimuli using a MEDOC PATHWAY Pain and Sensory Evaluation System (Medoc Limited, Ramat Yishai, Israel) which we connected with its 5 V TTL trigger-output to our EEG. The thermal probe for recording CHEPS delivers short heat bursts by increasing its temperature at a fixed rate of 70 °C/s and selectively activates A- and C-fiber nociceptors if an adequate peak temperature is chosen (Madsen et al. 2012; Rosner et al. 2018). The contact area of the CHEPS thermode is circular, with a diameter of 27 mm. We pseudo-randomized inter-stimulus interval between 8 and 12 s. We set our baseline temperature to 32 °C and our peak temperature to 54 °C. The peak temperature of 54 °C was chosen as it is above the 95% confidence interval of the heat pain threshold (HPT) in the QST reference data for feet stimulation in the age range of 15–35 years and should thus be perceived as painful by a healthy participant (Magerl et al. 2010). The mean values were 45.12 °C (40.42 °C; 49.81 °C) for males and 43.69 °C (38.20 °C; 49.19 °C) for females, with the 95% confidence interval in brackets (Magerl et al. 2010).

### Conditioned pain modulation (CPM) with PEP as readout

As a CS, we used a cold water bath, which we kept at 8 °C using ice packs. We applied the conditioning stimulus by having the participants submerge their non-dominant foot into the cold water. We asked the participants to rate the initial painfulness on the VAS right after inserting their foot into the cold water bath.

We then applied the TS on the same stimulation area as before (dorsal area of the dominant foot). We used our modified pinprick as outlined in the methods section for “PEP” with 12 stimuli in the same area. The only difference to the PEP was a reduced inter-stimulus interval which we randomized between 3 and 5 s and a summarized VAS rating after a twelfth stimulus (i.e., not every single stimulus was rated on the VAS). We recorded PEPs three times: (1) as a baseline recording before applying the CS, (2) 10 s after the participants submerged their foot into the cold water bath, while having the foot submerged during the whole application of the TS and (3) 60 s after the participants took their foot out of the cold water bath. Other settings and sequences were carried out as described in the methods section for “PEP”.

### EEG recording and pre-processing

The study took place with each participant sitting, in a quiet room. We asked the participant to place their dominant leg on a height-matched rack to allow for comfortable sitting during the whole study period. The investigators equipped the participants with a 64-channel (g.Tec g.SCARABEO, Guger Technologies, Schiedlberg, Austria) EEG cap (g.Tec g.GAMMAcap^2^). We decided to use active EEG electrodes to guarantee for an exceptionally low output impedance (below 1 Ω) and to minimize artifacts from movement of the electrode cables.

After recording the raw EEG in g.Tec’s proprietary.hdf5 format and storing it offline, we imported it into the MATLAB toolbox EEGLAB (Delorme and Makeig 2004). We down-sampled our EEGs to 256 Hz for the purpose of data reduction by utilizing EEGLAB’s function *pop_resample*. This function automatically applies the necessary low-pass filter. We applied a zero-phase bandpass filter by utilizing EEGLAB’s *eegfiltnew* function between 1 and 100 Hz and reduced line noise at 50 Hz with the EEGLAB CleanLine plugin. By visually inspecting our datasets, we removed corrupted channels (e.g., due to electrode popping) and interpolated them via spherical spline interpolation (Ferree 2006). On average, we rejected and interpolated 4 channels, while our area of interest (the Cz electrode) was never affected. By subsequently utilizing Artifact Subspace Reconstruction (ASR) with a tolerance parameter of 20, we applied an automated artifact rejection routine to our datasets to eliminate artifacts such as eye blinks and jaw clenching (Chang et al. 2018). As a last step, we epoched our data from − 1 s before the onset of each stimulus to +2 s after the onset of each stimulus.

We calculated the Event-Related Spectral Perturbation (ERSP) and the Inter-Trial Coherence (ITC) using EEGLAB’s *newtimef*-function with a divisive baseline from − 1 s to 0 s, a resolution in time of 400 points from − 1 s to +2 s and a frequency resolution of 200 points between the frequencies of 3 Hz and 100 Hz (Grandchamp and Delorme 2011; Herrmann et al. 2014). The function *newtimef*, which incorporates both a wavelet transform and a short-term Fourier transform, ran with 3 cycles at the lowest frequency (3 Hz) and 20 cycles at the highest frequency (100 Hz). Our electrode of interest for EEG analysis was the Cz (Iannetti et al. 2013; Granovsky et al. 2016; Anders et al. 2020). For comprehensibility, we show the ERSP between 3 and 45 Hz. In the ERSP, to be considered a response to a stimulus, we set a threshold for the changes in EEG power of [− 2 dB; 2 dB] and outlined areas that exceeded that threshold with a grey area in our figures.

### Fitness testing

We obtained the participants’ heart rates following the Astrand Rhyming Step test as a derivate of the submaximal heart rate of the participants. We additionally evaluated the heart rate at the beginning and at the end of the EEG measurement as an estimation of the resting heart rate in order to classify the performance level and cardiovascular capacity of the participants. The Astrand Rhyming Step test is a valid and reliable submaximal variation of the Harvard test (Marley and Linnerud 1976).

For the fitness test, the participants had to alternately climb a step with a gender-adjusted height for 5 min at a predetermined frequency of 90 steps per minute. We ensured the observance of the beat with an acoustic signal (metronome). The height for women was 33 cm, while for men this was 40 cm. Fifteen seconds after the measurement, we measured the participants’ heart rates manually at the wrist.

### Statistics

Due to our small sample size (Mishra et al. 2019) and because of its suitability for the analysis of EEG data (Maris and Oostenveld 2007), we adhered to a non-parametrical statistical approach throughout our analysis. In order to statistically evaluate possible differences between the groups (ERSP and VAS), we calculated the area under the receiver-operating characteristics (AUROC), together with 1000-fold bootstrapped 95% confidence intervals using the MES toolbox for MATLAB (Hentschke and Stüttgen 2011). An effect or difference can be considered significant if the 95% confidence interval for the AUROC does not include 0.5 (Hentschke and Stüttgen 2011). An AUROC = 0.5 indicates a completely random relationship, while an AUROC = 1 or AUROC = 0 indicates a perfect separation of the values between the groups, i.e., a perfect classifier (Jordan et al. 2010). According to the traditional point system, we reported effects presented as AUROC values as being excellent in the range of between 1 and 0.9, as good in the range of between 0.9 and 0.8, as fair in the range of between 0.8 and 0.7, as poor in the range of between 0.7 and 0.6 and as fail when they are below 0.6 (Tape 2001). For dependent data, we compared the relative change between two conditions versus a fixed value of 1 using the *auroc* function of the MES toolbox. For comprehensibility, we extracted the maximum ERSP and AUROC values out of the mentioned regions to extract the most objective ERSP response that was not dependent on the chosen window size.

To account for multiple comparisons, instead of a common approach of an alpha level adjustment, we applied a cluster-based approach, as it has been used in the literature both for 2-dimensional (Akeju et al. 2014; Kreuzer et al. 2020) and 3-dimensional (Lutz et al. 2022; Reiser et al. 2022) EEG data. We only reported results as being significant if they occurred in clusters of at least 4 × 4 adjacent significant pixels; this translates to a frequency range of 1.5 Hz and a time range of 15 ms.

We compared demographics between both groups (questionnaire scores and age) as well as the VAS scores using the Wilcoxon–Mann Whitney test, and binary questionnaire responses (Yes/No) using the Chi-squared test. Furthermore, we compared the VAS scores for the three different conditions during CPM testing (before, during and after the cold water bath) using the Friedman’s test. For post hoc testing, we utilized the Matlab function *multcompare*. For all median values, we show the 25% and the 75% percentiles in square brackets.

## Data Availability

The datasets generated during and/or analysed during the current study are available from the corresponding author on reasonable request.

## References

[CR1] Akeju O, Westover MB, Pavone KJ, Sampson AL, Hartnack KE, Brown EN, Purdon PL (2014). Effects of sevoflurane and propofol on frontal electroencephalogram power and coherence. Anesthesiology.

[CR2] Anders M, Anders B, Kreuzer M, Zinn S, Walter C (2020). Application of referencing techniques in EEG-based recordings of contact heat evoked potentials (CHEPS). Front Hum Neurosci.

[CR3] Anders B, Anders M, Kreuzer M (2022). Sensory testing and topical capsaicin can characterize patients with rheumatoid arthritis. Clin Rheumatol.

[CR4] Assa T, Geva N, Zarkh Y, Defrin R (2019). The type of sport matters: Pain perception of endurance athletes versus strength athletes. Eur J Pain.

[CR5] Bacchetti P, Deeks SG, McCune JM (2011). Breaking free of sample size dogma to perform innovative translational research. Sci Transl Med.

[CR6] Bannister K, Dickenson AH (2017). The plasticity of descending controls in pain: translational probing. J Physiol.

[CR7] Booth FW, Lees SJ (2006). Physically active subjects should be the control group. Med Sci Sports Exerc.

[CR8] Breuer C, Wicker P (2010). Sportökonomische Analyse der Lebenssituation von Spitzensportlern in Deutschland. Bundesinstitut für Sportwissenschaft.

[CR9] Buford TW, Manini TM (2010). Sedentary individuals as “controls” in human studies: the correct approach?. Proc Natl Acad Sci.

[CR10] Bumann A, Banzer W, Fleckenstein J (2020). Prevalence of biopsychosocial factors of pain in 865 sports students of the Dach (Germany, Austria, Switzerland) region - a cross-sectional survey. J Sports Sci Med.

[CR11] Chang CY, Hsu SH, Pion-Tonachini L, Jung TP (2018). Evaluation of artifact subspace reconstruction for automatic EEG artifact removal. Annu Int Conf IEEE Eng Med Biol Soc.

[CR12] Cohen MX (2011). It's about time. Front Hum Neurosci.

[CR13] Delorme A, Makeig S (2004). EEGLAB: an open source toolbox for analysis of single-trial EEG dynamics including independent component analysis. J Neurosci Methods.

[CR14] Fabig SC, Kersebaum D, Lassen J (2021). A modality-specific somatosensory evoked potential test protocol for clinical evaluation: a feasibility study. Clin Neurophysiol.

[CR15] Farahbakhsh F, Rostami M, Noormohammadpour P (2018). Prevalence of low back pain among athletes: a systematic review. J Back Musculoskelet Rehabil.

[CR16] Ferree TC (2006). Spherical splines and average referencing in scalp electroencephalography. Brain Topogr.

[CR17] Fett D, Trompeter K, Platen P (2017). Back pain in elite sports: a cross-sectional study on 1114 athletes. PLoS One.

[CR18] Geisler M, Ritter A, Herbsleb M, Bär K-J, Weiss T (2021). Neural mechanisms of pain processing differ between endurance athletes and nonathletes: a functional connectivity magnetic resonance imaging study. Hum Brain Mapp.

[CR19] Geva N, Defrin R (2013). Enhanced pain modulation among triathletes: a possible explanation for their exceptional capabilities. Pain.

[CR20] Glover GH (2011). Overview of functional magnetic resonance imaging. Neurosurg Clin N Am.

[CR21] Grandchamp R, Delorme A (2011). Single-trial normalization for event-related spectral decomposition reduces sensitivity to noisy trials. Front Psychol.

[CR22] Granovsky Y, Anand P, Nakae A, Nascimento O, Smith B, Sprecher E, Valls-Solé J (2016). Normative data for Aδ contact heat evoked potentials in adult population: a multicenter study. Pain.

[CR23] Hartley C, Duff EP, Green G, Mellado GS, Worley A, Rogers R, Slater R (2017). Nociceptive brain activity as a measure of analgesic efficacy in infants. Sci Transl Med.

[CR24] Hentschke H, Stüttgen MC (2011). Computation of measures of effect size for neuroscience data sets. Eur J Neurosci.

[CR25] Herrmann CS, Rach S, Vosskuhl J, Strüber D (2014). Time-frequency analysis of event-related potentials: a brief tutorial. Brain Topogr.

[CR26] Hüllemann P, Nerdal A, Sendel M, Dodurgali D, Forstenpointner J, Binder A, Baron R (2019). Cold-evoked potentials versus contact heat-evoked potentials—methodological considerations and clinical application. Eur J Pain.

[CR27] Iannetti GD, Hughes NP, Lee MC, Mouraux A (2008). Determinants of laser-evoked EEG responses: pain perception or stimulus saliency?. J Neurophysiol.

[CR28] Iannetti GD, Baumgärtner U, Tracey I, Treede RD, Magerl W (2013). Pinprick-evoked brain potentials: a novel tool to assess central sensitization of nociceptive pathways in humans. J Neurophysiol.

[CR29] Jordan D, Steiner M, Kochs EF, Schneider G (2010). A program for computing the prediction probability and the related receiver operating characteristic graph. Anesth Analg.

[CR30] Jutzeler CR, Rosner J, Rinert J, Kramer JL, Curt A (2016). Normative data for the segmental acquisition of contact heat evoked potentials in cervical dermatomes. Sci Rep.

[CR31] Kim SG, Richter W, Uğurbil K (1997). Limitations of temporal resolution in functional MRI. Magn Reson Med.

[CR32] Kreuzer M, Stern MA, Hight D, Berger S, Schneider G, Sleigh JW, García PS (2020). Spectral and entropic features are altered by age in the electroencephalogram in patients under sevoflurane anesthesia. Anesthesiology.

[CR33] Latremoliere A, Woolf CJ (2009). Central sensitization: a generator of pain hypersensitivity by central neural plasticity. J Pain.

[CR34] Legrain V, Iannetti GD, Plaghki L, Mouraux A (2011). The pain matrix reloaded: a salience detection system for the body. Prog Neurobiol.

[CR35] Lewis GN, Rice DA, McNair PJ (2012). Conditioned pain modulation in populations with chronic pain: a systematic review and meta-analysis. J Pain.

[CR36] Lutz R, Müller C, Dragovic S (2022). The absence of dominant alpha-oscillatory EEG activity during emergence from delta-dominant anesthesia predicts neurocognitive impairment- results from a prospective observational trial. J Clin Anesth.

[CR37] Madsen CS, Johnsen B, Fuglsang-Frederiksen A, Jensen TS, Finnerup NB (2012). The effect of nerve compression and capsaicin on contact heat-evoked potentials related to Aδ- and C-fibers. Neuroscience.

[CR38] Magerl W, Fuchs PN, Meyer RA, Treede RD (2001). Roles of capsaicin-insensitive nociceptors in cutaneous pain and secondary hyperalgesia. Brain.

[CR39] Magerl W, Krumova EK, Baron R, Tölle T, Treede RD, Maier C (2010). Reference data for quantitative sensory testing (QST): refined stratification for age and a novel method for statistical comparison of group data. Pain.

[CR40] Main CJ (2016). Pain assessment in context: a state of the science review of the McGill pain questionnaire 40 years on. Pain.

[CR41] Maris E, Oostenveld R (2007). Nonparametric statistical testing of EEG- and MEG-data. J Neurosci Methods.

[CR42] Marley WP, Linnerud AC (1976). Astrand-ryhming step test norms for college students. Br J Sports Med.

[CR43] McDougall J, Jutzeler CR, Scott A, Crocker PRE, Kramer JLK (2020). Conditioned pain modulation in elite athletes: a systematic review and meta-analysis. Scand J Pain.

[CR44] Mishra P, Pandey CM, Singh U, Keshri A, Sabaretnam M (2019). Selection of appropriate statistical methods for data analysis. Ann Card Anaesth.

[CR45] Murrell JC, Johnson CB (2006). Neurophysiological techniques to assess pain in animals. J Vet Pharmacol Ther.

[CR46] Nijs J, George SZ, Clauw DJ (2021). Central sensitisation in chronic pain conditions: latest discoveries and their potential for precision medicine. Lancet Rheumatol.

[CR47] Nir RR, Yarnitsky D (2015). Conditioned pain modulation. Curr Opin Support Palliat Care.

[CR48] Özgül ÖS, Maier C, Enax-Krumova EK, Vollert J, Fischer M, Tegenthoff M, Höffken O (2017). High test-retest-reliability of pain-related evoked potentials (PREP) in healthy subjects. Neurosci Lett.

[CR49] Pescatello LSARRDTPDACoSMLW, Wilkins (2014) ACSM's guidelines for exercise testing and prescription. Wolters Kluwer Health/Lippincott Williams & Wilkins, Philadelphia

[CR50] Pettersen SD, Aslaksen PM, Pettersen SA (2020). Pain processing in elite and high-level athletes compared to non-athletes. Front Psychol.

[CR51] Raja SN, Carr DB, Cohen M (2020). The revised international association for the study of pain definition of pain: concepts, challenges, and compromises. Pain.

[CR52] Reiser J, Kreuzer M, Werner J (2022). Nociception-induced changes in electroencephalographic activity and FOS protein expression in piglets undergoing castration under isoflurane anaesthesia. Animals (basel).

[CR53] Rolke R, Baron R, Maier C (2006). Quantitative sensory testing in the german research network on neuropathic pain (DFNS): standardized protocol and reference values. Pain.

[CR54] Ronga I, Valentini E, Mouraux A, Iannetti GD (2013). Novelty is not enough: laser-evoked potentials are determined by stimulus saliency, not absolute novelty. J Neurophysiol.

[CR55] Rosner J, Hostettler P, Scheuren PS (2018). Normative data of contact heat evoked potentials from the lower extremities. Sci Rep.

[CR56] Roussel NA, Nijs J, Meeus M, Mylius V, Fayt C, Oostendorp R (2013). Central sensitization and altered central pain processing in chronic low back pain: fact or myth?. Clin J Pain.

[CR57] Scheef L, Jankowski J, Daamen M (2012). An fMRI study on the acute effects of exercise on pain processing in trained athletes. Pain.

[CR58] Senba E, Kami K (2017). A new aspect of chronic pain as a lifestyle-related disease. Neurobiol Pain.

[CR59] Sluka KA, Frey-Law L, Hoeger Bement M (2018). Exercise-induced pain and analgesia? Underlying mechanisms and clinical translation. Pain.

[CR60] Sommer C (2016). Exploring pain pathophysiology in patients. Science.

[CR61] Swann C, Moran A, Piggott D (2015). Defining elite athletes: Issues in the study of expert performance in sport psychology. Psychol Sport Exerc.

[CR62] Tape TG (2001). Interpretation of Diagnostic Tests. Ann Intern Med.

[CR63] Tesarz J, Schuster AK, Hartmann M, Gerhardt A, Eich W (2012). Pain perception in athletes compared to normally active controls: a systematic review with meta-analysis. Pain.

[CR64] Tesarz J, Gerhardt A, Schommer K, Treede RD, Eich W (2013). Alterations in endogenous pain modulation in endurance athletes: an experimental study using quantitative sensory testing and the cold-pressor task. Pain.

[CR65] Treede RD, Rief W, Barke A (2019). Chronic pain as a symptom or a disease: the iasp classification of chronic pain for the international classification of diseases (ICD-11). Pain.

[CR66] van den Broeke EN, Mouraux A, Groneberg AH, Pfau DB, Treede R-D, Klein T (2015). Characterizing pinprick-evoked brain potentials before and after experimentally induced secondary hyperalgesia. J Neurophysiol.

[CR67] van den Broeke EN, de Vries B, Lambert J, Torta DM, Mouraux A (2017). Phase-locked and non-phase-locked EEG responses to pinprick stimulation before and after experimentally-induced secondary hyperalgesia. Clin Neurophysiol.

[CR68] Ziegler EA, Magerl W, Meyer RA, Treede RD (1999). Secondary hyperalgesia to punctate mechanical stimuli. Central sensitization to A-fibre nociceptor input. Brain.

